# Fermentative profile and bacterial community structure of whole-plant triticale silage (*Triticosecale Wittmack*) with or without the addition of *Streptococcus bovis* and *Lactiplantibacillus plantarum*

**DOI:** 10.1128/msphere.00894-24

**Published:** 2025-01-28

**Authors:** Yujie Niu, Yaya Guo, Rongzheng Huang, Junli Niu, Yan Wang, Peng Zhang, Qicheng Lu, Wenju Zhang

**Affiliations:** 1 Animal nutrition and feed science, College of Animal Science and Technology, Shihezi University, Shihezi, China; University of South Africa, Florida, Johannesburg, Gauteng, South Africa

**Keywords:** triticale, silage, inoculants, fermentation, bacterial community

## Abstract

This study aimed to investigate the effects of *Streptococcus bovis* and *Lactiplantibacillus plantarum* on the chemical composition, fermentation characteristics, bacterial communities, and predicted metabolic pathways of whole-plant triticale silage (*Triticosecale Wittmack*). Fresh triticale harvested at the milk stage was ensiled in sterile distilled water (CON), *Streptococcus bovis* (ST), *Lactiplantibacillus plantarum* (LP), and a combination of *S. bovis* and *L. plantarum* (LS) for 3, 7, 15, and 30 days. During ensiling, the pH and water-soluble carbohydrate (WSC) content in the inoculated groups was significantly lower than those in the CON group (*P* < 0.05), especially in the LS group (*P* < 0.05). After 7 days of ensiling, the ST and LS groups had lower (*P* < 0.05) starch content and higher (*P* < 0.05) concentrations of lactic acid (LA) and acetic acid (AA). Inoculation with *S. bovis* and *L. plantarum*, either alone or in combination, increased the abundance of *Lactobacillus* and *Pediococcus* while reducing the abundance of *Weissella*, *Rosenbergiella*, *Pantoea*, and *Enterobacter*. Metabolic prediction analysis indicated that inoculation with *S. bovis* enhanced starch and sucrose metabolism during the early stages of ensiling. The abundance of *Streptococcus* positively correlated with LA (*P* < 0.05) and AA concentration but negatively correlated with starch content, pH, and propionic acid (PA) concentration (*P* < 0.05). *S. bovis* inoculation increased starch hydrolysis and carbohydrate metabolism during the early stages. *S. bovis* and *L. plantarum* synergistically improved the fermentation characteristics and bacterial communities of triticale silage. Therefore, *S. bovis* can be used as an additive or for a fast start-up agent to improve silage fermentation quality.

**IMPORTANCE:**

Ensiling is a widely used method for preserving fresh forage. Silage quality is determined by the chemical and microbial composition. Studies have shown that *S. bovis* grew faster than commercial species, thereby creating advantages for other lactic acid bacteria during ensiling. Therefore, we believe that *S. bovis* and *L. plantarum* can synergistically improve the fermentation characteristics and bacterial community in silage. However, few studies use high-throughput methods to explain the impact of *S. bovis* on silage fermentation. Results showed that *S. bovis* significantly affected the fermentation parameters, bacterial community, and metabolic characteristics of triticale silage. *S. bovis* and *L. plantarum* synergistically accelerated the fermentation, reducing pH and WSC while increasing lactic acid and acetic acid concentrations in the early stages of ensiling. Additionally, co-inoculation increased the abundance of *Lactobacillus* and *Pediococcus* and carbohydrate metabolism. This study emphasizes the synergistic role of *S. bovis* and *L. plantarum* in enhancing triticale silage quality, providing scientific support for novel silage additives.

## INTRODUCTION

Triticale (*Triticosecale Wittmack*), a hybrid of wheat (*Triticum* spp.) and rye (*Secale cereale*), belongs to the grass family Poaceae (1). It combines the grain quality of wheat with the robust vitality and high forage yield of rye, making triticale grains suitable for human consumption and whole plants valuable as livestock feed (2). Moreover, the yield of whole-plant triticale can reach 4.5 kg/m^2^, and the protein (9.4%–16.5%), lysine (0.31%–0.71%), and starch (57.73%–66.50%) content of triticale grain make it an attractive ruminant forage (3–5). However, the hollow stem structure of triticale may make it more susceptible to aerobic degradation during ensiling, potentially affecting the preservation of nutrients (6).

Ensiling is a common technique used in the livestock industry to preserve fresh forage, prevent nutrient degradation, and maintain feed palatability. In anaerobic environments, lactic acid bacteria (LAB) proliferate rapidly, producing organic acids that lower pH and inhibit undesirable bacteria (6). However, natural forage fermentation often leads to nutrient loss and low-quality silage (7). Therefore, additives, such as chemicals, enzymes, LAB, and non-LAB species, are commonly used to enhance silage quality (6). LAB inoculants, such as *Lactiplantibacillus plantarum*, are commonly used as additives in silage. *L. plantarum* inoculants typically have lower pH, ammonia-N (AN), and dry matter (DM) loss but higher LA concentrations than untreated silage (8). However, *L. plantarum* primarily improves fermentation byproducts and inhibits the growth of harmful bacteria rather than actively enhancing starch breakdown, which could be important for improving the quality of silage from high-starch crops such as triticale.

Here, *Streptococcus bovis* (*S. bovis*) may provide additional benefits. *S. bovis*, a lactate-producing bacterium found in the rumen, has a rapid proliferation rate (about 30% faster than other LAB like *Leuconostoc mesenteroides* and *Levilactobacillus brevis*) and can hydrolyze starch into glucose and other fermentable sugars, thereby enhancing the production of LA during ensiling (9, 10). Ferreira et al. (11) reported that *S. bovis* treatment of elephant grass silage reduced pH and AN, decreased gas and effluent loss, and improved DM recovery compared with untreated or *Enterobacter faecalis*-treated samples. Zanine et al. (9) demonstrated that the application of *S. bovis* decreased crude protein (CP) loss in Tanzania grass silage. Generally, whole-plant corn, wheat, and other grain feeds are rich in starch, and LAB additives cannot be efficiently utilized during ensiling. However, *S. bovis* can decompose starch into glucose and other carbohydrates and utilize them during fermentation to produce additional LA (10). Furthermore, *S. bovis* HC5 inhibits the growth of undesired bacteria such as *yeasts*, *Escherichia*, and *Salmonella* by producing a bacteriocin similar to monensin (12, 13).

While much has been studied about individual LAB inoculants like *L. plantarum* and *S. bovis*, there is still a gap in the understanding of how the combination of these two bacteria could enhance the silage fermentation. Combining *S. bovis* with *L. plantarum* may have a synergistic effect by not only improving the fermentation efficiency through starch hydrolysis but also suppressing undesirable microbial populations through the antimicrobial properties of both bacteria. Therefore, we hypothesized that *S. bovis* and *L. plantarum* could modulate the chemical composition and fermentation products by changing the bacterial community in silage and may have a synergistic effect when inoculated together.

## MATERIALS AND METHODS

### Bacterial safety testing

#### Hemolytic activity testing of *S. bovis*

According to traditional hemolytic testing methods (14), 7% sheep blood agar (Yuanye Biological Co., LTD, Shanghai, China) was used. Isolated *S. bovis* strains were streaked onto the agar, with *Staphylococcus aureus* (ATCC 25923) serving as a positive control. The inoculated plates were incubated at 37°C for 24 h. Hemolytic patterns around the colonies were observed and recorded as follows: α-hemolysis (partial hemolysis): a green or gray-green zone around the colony; β-hemolysis (complete hemolysis): a clear zone around the colony; γ-hemolysis (no hemolysis): no color change or clear zone around the colony.

#### Antimicrobial susceptibility evaluation of *S. bovis*

The Kirby-Bauer disk diffusion method was used for antimicrobial susceptibility testing (15). *S. bovis* was cultured on Mueller-Hinton agar. Prepare antibiotic discs using selected antimicrobial agents. The antibiotic discs were evenly placed on the inoculated agar surface. The plates were incubated at 37°C for 24 h, and the inhibition zone diameters were measured. According to CLSI standards (2018), the strain’s sensitivity to each antibiotic was classified as follows: sensitive (S): inhibition zone diameter larger than the standard value; intermediate (I): inhibition zone diameter within the standard range; resistant (R): inhibition zone diameter smaller than the standard value.

#### Acute oral toxicity test of *S. bovis*

This study followed the Organization for Economic Co-operation and Development (OECD) test guidelines (16). Healthy male and female mice (6–8 weeks old, 24–26 g) were randomly assigned to experimental and control groups, with 10 mice per group (5 males and 5 females). Mice in the treatment group were orally gavaged with 0.2 mL of *S. bovis* at 1 × 109 cfu/mL daily for 30 days, while the CON received 0.2 mL of sterile normal saline. The mice were provided with sufficient feed and water *ad libitum*. Clinical signs and mortality were observed daily, and food intake and BW were monitored weekly. At the end of the gavage period, the mice were humanely euthanized.

### Forage and ensiling

Triticale (*Triticosecale wittmack*; Varitety: Shida No. 1) was planted in an experimental field in Changji, Xinjiang, China (44°18′N, 86°27′E, altitude 370 m; average annual temperature 10.0°C, average annual precipitation 167 mm) and harvested at the milk stage on 6 June 2022. The initial height of the fresh grass stubble was 5–10 cm. After wilting for 2 h, the triticale was cut into 2 cm pieces when its moisture content was reduced to approximately 65%. The experiment included four groups: CON (sterile distilled water), ST (*S. bovis* isolated from the rumen of cattle, OQ812187), LP (commercial *L. plantarum*; Shandong Zhongke Jiayi Bioengineering Co., Ltd., Weifang, Shandong, China), and LS (*S. bovis + L. plantarum*). The LS group contained an equal ratio (1:1) of both bacterial types. One thousand grams of fresh samples was packed into 2 L silo, after compacted and vacuum sealed to obtain 500 kg/m³ ensiling densities. The bacteria suspension with a concentration of 10^6^ cfu/g FM in each treatment was evenly sprayed on the ground fresh samples using a spray bottle, and the equal volume of sterile distilled water was sprayed on the CON group (17). The silage samples were stored indoors at 21–24°C. Quintuplicate samples from each treatment were opened after 3, 7, 15, and 30 days of ensiling, respectively.

### Chemical composition analysis

Samples were tested for DM content by drying in a forced-draft oven at 60°C for 48 h. The dried samples were then ground in a mill and passed through a 1 mm screen for chemical composition analysis (18). The WSC content was determined using the sulfate-anthrone method (19). The CP content was determined using a Kjeldahl nitrogen analyzer (Kjeltec 8200, FOSS Analytical A/S, Hillerød, Denmark) following the AOAC procedures (20). The starch content was measured using a starch content assay kit (BC0700, Solarbio, Beijing, China). Acid detergent fiber (ADF) and neutral detergent fiber (NDF) contents were analyzed using the method described by Van Soest (18).

### Fermentation characteristics analysis

Twenty grams of fresh sample was mixed with 180 mL of sterile distilled water and thoroughly homogenized. The mixture was stored at 4°C overnight. Before the measurement, the mixture was filtered through four layers of sterile gauze. The pH was determined using a portable pH meter (WTW pH 3110, Xylem Inc. Munich, Germany), and then the mixture was centrifuged at 12,000 × *g* for 10 min at 4°C. The filtrate was used to measure the AN (21). The remaining samples were filtered through a 0.22 µm aqueous phase filter membrane. The filtered samples were then used to determine organic acid concentrations using the HPLC 1290 (Agilent Technologies, Inc., Santa Clara, CA, USA) system with a C18 column (150 mm × 4.6 mm, FMF-5559-EONU FLM Scientific Instruments Co., LTD., Guangzhou, China). The mobile phase comprised Na_2_HPO_4_ (1 mM) with a flow rate of 0.6 mL·min^−1^, a column oven temperature of 50°C, and an injection volume of 20 µL (22).

### Bacterial community analysis

Based on the chemical composition and fermentation characteristics of the silage, samples from days 0 (FM), 7, and 30 were selected for bacterial community analysis by next-generation sequencing (NGS). Before further analysis, total bacterial genomic DNA was extracted using the DNeasy PowerSoil Kit (QIAGEN, Inc., Netherlands) according to the manufacturer’s instructions and stored at −20°C. The quantity and quality of extracted DNAs were measured using a NanoDrop ND-1000 spectrophotometer (Thermo Fisher Scientific, Waltham, MA, USA) and agarose gel electrophoresis.

Sequencing was performed using the Illumina MiSeq platform with MiSeq Reagent Kit v3 (Shanghai Personal Biotechnology Co., Ltd., Shanghai, China). The Quantitative Insights into Microbial Ecology pipeline (QIIME, v1.8.0) was used to process the sequencing data. For the analysis of bacterial community data, Dada2 (23) was used to eliminate primers, filter quality, denoise, splice, and chimeras to produce feature sequences. Bacterial community data were examined at both the phylum and genus levels using the Silva database (Release132, http://www. arb-silva. de). Bacterial community functionality was predicted using Phylogenetic Investigation of Communities by Reconstruction of Unobserved States (PICRUSt2; https://github.com/picrust/picrust2/wiki/; Version 2.3.0) based on the abundance of marker gene sequences in the samples (24). The annotated results were then “mapped” to gene families in the KEGG database (https://www.kegg.jp/) to obtain metabolic pathway abundance data.

### Statistical analysis

The effects of inoculants and ensiling days on the chemical composition and fermentation characteristics of triticale silage were analyzed according to the Wang et al. (25). The experiment employed a completely randomized design with four treatments, four ensiling days, and five replicates per treatment. The chemical composition and fermentation characteristics were analyzed using two-way analysis of variance (ANOVA) with the Statistical Package for the Social Sciences (SPSS version 22.0; SPSS Inc., Chicago, IL, USA). A factorial design with four treatments and four ensiling days was used.


Yij=μ+Di+Tj+(D×T)ij+eij,

*Yij* represents the dependent variable, μ is the overall mean, *Ti* is the effect of treatment, *Dj* is the effect of ensiling day, (*T* × *D*)*ij* is the effect of interaction between treatment and ensiling day, and *eij* is the residual effect.

Statistical analysis was performed using the Wilcoxon test to analyze the chemical compositions, fermentation characteristics, and bacterial composition between the different ensiling days. The polynomial orthogonal contrasts (linear and quadratic) were used to determine the response to ensiling day at different inoculants of triticale. Statistical difference was measured by Tukey’s multiple comparison. Statistical significance was set at *P* < 0.05.

## RESULTS

### Safety analysis of *S. bovis*

As shown in Fig. S1, *S. bovis* exhibited γ-hemolysis (no hemolytic activity) after 24 h of incubation on blood agar. The antibiotic susceptibility results indicated that *S. bovis* showed intermediate resistance to enrofloxacin, cefalotin, and neomycin; resistance to levofloxacin and tetracycline; and sensitivity to vancomycin, clindamycin, gentamicin, and five other antibiotics (Table S1). Acute oral toxicity tests in mice revealed no significant differences in food intake, daily weight gain, clinical signs, or mortality between the EXP and the CON (Tables S2 and S3). Overall, these experimental results suggest that *S. bovis* is safe for both the environment and animals.

### Chemical compositions of fresh triticale

The chemical composition of fresh whole-plant triticale is presented in Table 1. The pH of triticale before ensiling was 6.30. The DM content was 371 g/kg of FM. WSC, starch, CP, NDF, and ADF contents were 98.71, 136, 101, 592, and 272 g/kg of DM, respectively. The microorganism counts for LAB, yeast, mold, and aerobic bacteria were 3.66, 4.78, 4.12, and 8.54 log_10_ cfu/g of FM, respectively.

**TABLE 1:** The chemical compositions of fresh triticale^*a*^

Items	Content
DM, g/kg of FM	371
WSC, g/kg of DM	98.7
Starch, g/kg of DM	136
CP, g/kg of DM	101
NDF, g/kg of DM	592
ADF, g/kg of DM	272
pH	6.30
Microbial populations (log_10_ cfu/g of FM)	
Lactic acid bacteria	3.66
Yeast	4.78
Mold	4.12
Aerobic bacteria	8.54

^*a*^ FM, fresh matter; DM, dry matter; CP, crude protein; NDF, neutral detergent fiber; ADF, acid detergent fiber; WSC, water-soluble carbohydrate; CFU, colony-forming unit.

### Chemical composition of triticale silage

The samples were collected at four-time points to study the effect of the inoculant and ensiling day on the chemical composition of the triticale silage. The chemical compositions after 3, 7, 15, and 30 days of ensiling are listed in Table 2. The statistical results indicated that the inoculant had a significant (*P* < 0.001) effect on WSC and starch content, and the ensiling day had a significant (*P* < 0.001) effect on DM, WSC, CP, and starch content. On days 3 and 7, the CON group exhibited the highest WSC content, whereas the LS group exhibited the lowest content (*P* < 0.05). On days 15 and 30, a further decline in the WSC content was observed across all treatments, with the LS group consistently showing the lowest levels. Notably, on day 7, the ST and LS groups had significantly lower starch content than the CON and LP groups (*P* < 0.05). Specifically, the starch hydrolysis rate in the ST group increased by 9.16% and 4.98% compared to the CON and LP groups, respectively; in the LS group, the starch hydrolysis rate increased by 10.82% and 6.58% compared to the CON and LP groups, respectively.

**TABLE 2:** Effects of inoculant and days on chemical composition of triticale silage

Items	Days	Treatment				SEM	*P*-value		
		CON	ST	LP	LS		T	D	T × D
DM, g/kg of FM	3	360^A^	361^A^	361^A^	361^A^	5.082	0.073	< 0.001	0.418
	7	359^B^	355^B^	357^B^	352^B^				
	15	350^C^	348^C^	345^C^	348^C^				
	30	315^D^	331^D^	328^D^	323^D^				
WSC, g/kg of DM	3	76.1^Aa^	72.5^Ab^	72.7^Ab^	66.1^Ac^	1.519	< 0.001	< 0.001	0.155
	7	67.1^Ba^	63.1^Ab^	65.3^Aab^	55.0^Bc^				
	15	55.2^Ca^	56.8^Ba^	52.5^Bb^	42.6^Cc^				
	30	46.4^Da^	41.5^Ca^	38.1^Cab^	30.9^Db^				
Starch, g/kg of DM	3	127^Aa^	122^Ab^	126^Aab^	122^Aab^	1.718	< 0.001	< 0.001	0.269
	7	122^ABa^	112^Bc^	117^Bb^	110^Bc^				
	15	115^BCa^	102^Cc^	110^Cab^	107^Cbc^				
	30	110^Ca^	102^Cb^	105^BCb^	103^Cb^				
CP, g/kg of DM	3	94.8^A^	94.4^A^	95.2^A^	94.3^A^	1.763	0.454	< 0.001	0.079
	7	89.1^B^	93.5^A^	88.5^B^	90.5^B^				
	15	87.7^BC^	83.7^B^	87.6^B^	86.8^C^				
	30	84.9^C^	83.3^B^	85.3^B^	83.8^C^				

^*a*^ FM, fresh matter; CP, crude protein; WSC, water-soluble carbohydrate; CON, control (treated with distilled water); ST, inoculated with *S. bovis*; LP, inoculated with *L. plantarum*; LS, inoculated with *S. bovis* and *L. plantarum*; A−D, values with different capital letters within the same column indicate significant differences between ensiling days in the same treatment (*P* < 0.05); a−c, values with different lowercase letters with the same row indicate significant differences among treatments on the same ensiling day (*P* < 0.05); SEM, standard error of means; T, the effect of treatment; D, the effect of fermentation days; T × D, the interaction between treatment and fermentation day.

### Fermentation characteristics of triticale silage

Fermentation characteristics were analyzed by monitoring the pH, LA, AA, PA, and AN concentrations at 3, 7, 15, and 30 days (Table 3). The interaction between the treatments and ensiling days significantly (*P* < 0.05) affected pH, LA, and AA concentrations. PA concentration was only affected by the ensiling days (*P* < 0.05), and AN concentration was affected by the treatments (*P* < 0.05).

**TABLE 3:** Effects of inoculant and days on fermentation characteristics of triticale silage^*a*^

Items	Days	Treatments				SEM	*P*-value		
		CON	ST	LP	LS		D	T	D × T
pH	3	4.12^Aa^	4.05^Ab^	4.03^Ab^	3.98^Ac^	0.013	< 0.001	< 0.001	0.033
	7	3.93^B^	3.89^B^	3.91^B^	3.90^B^				
	15	3.92^Ba^	3.85^Cb^	3.83^Cb^	3.78^Cc^				
	30	3.80^Ca^	3.74^Db^	3.73^Db^	3.71^Db^				
LA, g/kg of DM	3	53.6^Cb^	62.2^Ca^	54.0^Db^	60.9^Da^	3.619	0.001	< 0.001	0.027
	7	69.3^Bb^	77.9^Ba^	69.6^Cb^	76.6^Ca^				
	15	78.0^Bb^	83.8^Bb^	89.6^Bab^	96.7^Ba^				
	30	97.6^Ab^	99.1^Ab^	105^Aab^	112^Aa^				
AA, g/kg of DM	3	3.55^Cbc^	6.06^Da^	2.76^Dc^	5.40^Dab^	0.653	< 0.001	< 0.001	0.005
	7	9.07^Bab^	11.3^Ca^	8.18^Cc^	10.7^Ca^				
	15	11.3^B^	13.3^B^	12.8^B^	13.3^B^				
	30	19.9^Ac^	22.7^Ab^	24.1^Aab^	25.1^Aa^				
PA, g/kg of DM	3	1.58^B^	1.06^D^	2.17^B^	1.32^D^	0.345	0.206	< 0.001	0.605
	7	3.26^B^	2.41^C^	3.62^B^	2.44^C^				
	15	3.52^B^	3.51^B^	3.64^B^	4.40^B^				
	30	7.04^A^	6.45^A^	6.86^A^	7.50^A^				
BA, g/kg of DM	3	ND	ND	ND	ND	-	-	-	-
	7	ND	ND	ND	ND				
	15	ND	ND	ND	ND				
	30	ND	ND	ND	ND				
AN, g/kg of DM	3	0.214^Dc^	0.253^Dab^	0.228^Dbc^	0.271^Da^	0.026	0.091	< 0.001	0.726
	7	0.524^Cbc^	0.567^Cab^	0.557^Cb^	0.607^Ca^				
	15	0.917^B^	0.888^B^	0.877^B^	0.921^B^				
	30	1.27^A^	1.26^A^	1.31^A^	1.31^A^				
LA/AA	3	17.3^Aab^	10.4^Ab^	20.0^Aa^	11.5^Ab^	1.138	< 0.001	0.003	0.004
	7	7.72^Bab^	6.90^Bb^	8.53^Ba^	7.19^Bb^				
	15	7.12^B^	6.29^B^	7.03^BC^	7.30^B^				
	30	4.92^B^	4.37^C^	4.34^C^	4.47^C^				


^*a*^ FM, fresh matter; DM, dry matter; AN, ammonia nitrogen; LA/AA, lactic acid to acetic acid ratio; CON, control (treated with distilled water); ST, inoculated with *S. bovis*; LP, inoculated with *L. plantarum*; LS, inoculated with *S. bovis* and *L. plantarum*; A−D, values with different capital letters within the same column indicate significant differences between ensiling days in the same treatment (*P* < 0.05); a−c, values with different lowercase letters with the same row indicate significant differences among treatments on the same ensiling day (*P* < 0.05); SEM, standard error of means; T, the effect of treatment; D, the effect of fermentation days; T × D, the interaction between treatment and fermentation day.

On day 3, the LS group exhibited the lowest pH (3.98), which was significantly higher than that of the other groups (*P* < 0.05). The lowest pH value was observed in the LS group on day 30. Correspondingly, the LS group had the highest LA concentration (*P* < 0.05). During fermentation, the DM and WSC contents were (*P* < 0.05) decreasing continuously, whereas the LA, AA, PA, and AN concentrations continuously (*P* < 0.05) increased. Although there were no significant (*P* > 0.05) differences in DM content among the groups, the ST and LS groups showed higher (*P* < 0.05) AN concentrations than the CON group on day 3. Butyric acid (BA) was undetectable in the silage.

### Bacterial community of triticale silage

**Fig 1:**
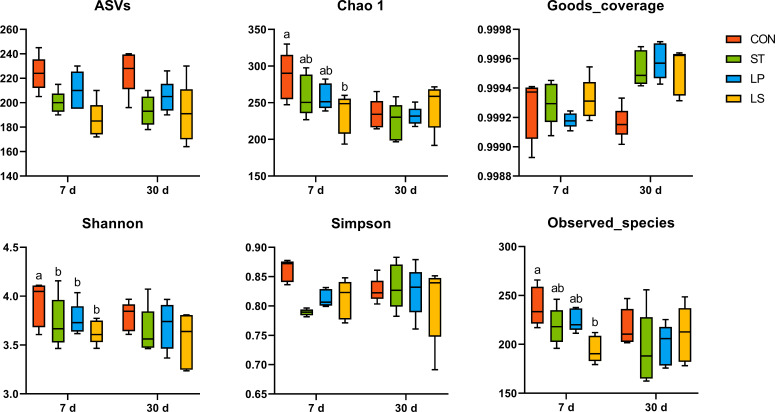
Amplicon sequence variants (ASVs) and alpha diversity of the bacterial community analysis for silage in fresh materials and triticale silage after 7 and 30 days of ensiling; FM, fresh matter CON, control (treated with distilled water); ST, inoculated with *S. bovis*; LP, inoculated with *L. plantarum*; LS, inoculated with *S. bovis* and *L. plantarum.*

The results of amplicon sequence variants (ASVs) and alpha diversity of the bacterial community analysis for silage are presented in Fig. 1. The ASVs of the samples decreased after ensiling, suggesting that anaerobic fermentation reduced the microbial diversity of fresh triticale. On day 7, the observed species and Shannon indices in the ST, LP, and LS groups were significantly lower than those in the CON group (*P* < 0.05). Notably, the observed species, Shannon indices, and Chao1 indices in the LS group were the lowest compared to the other groups. On day 30, there were no significant differences in alpha diversity indices between the groups.

**Fig 2:**
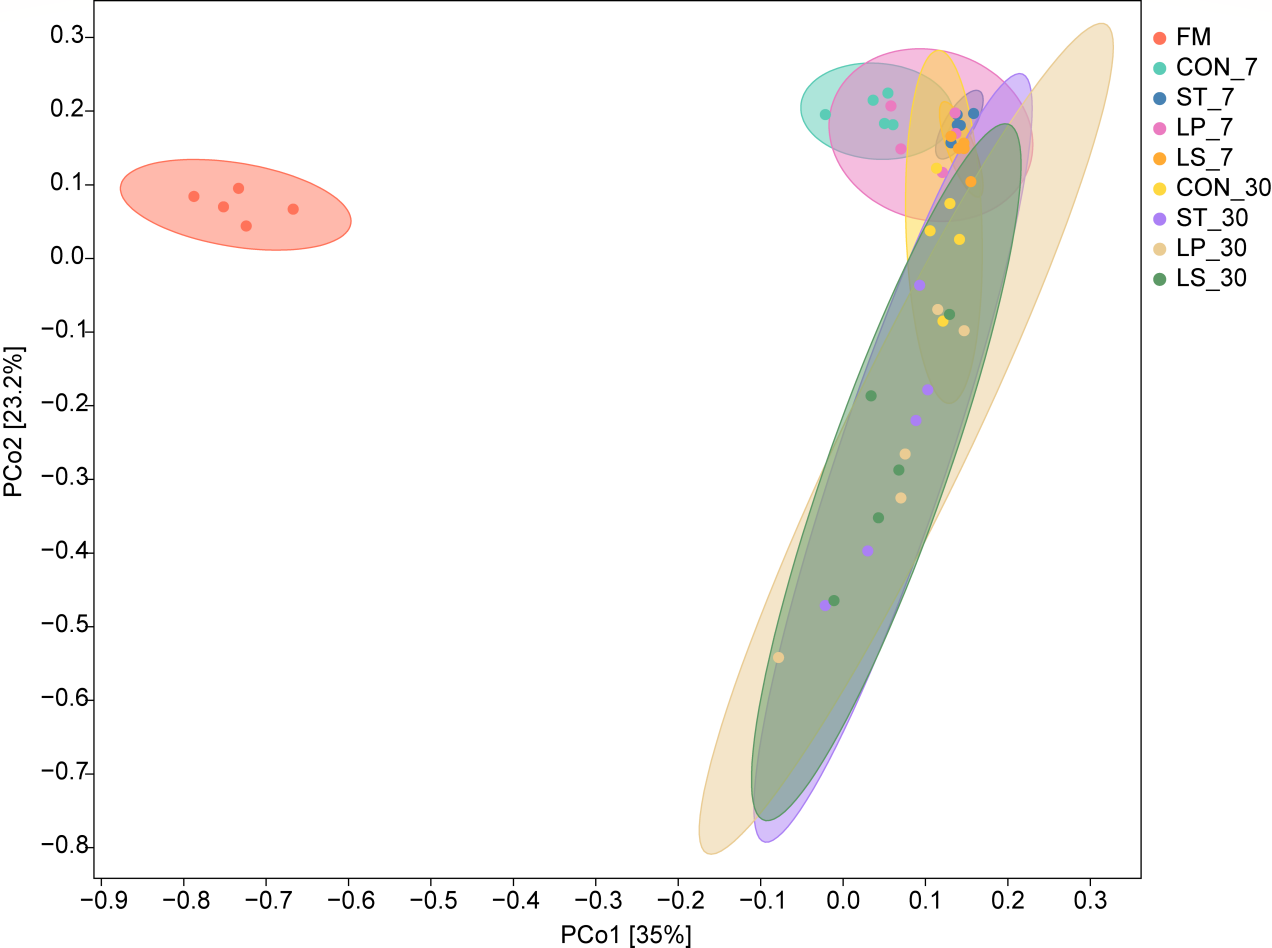
Principal coordinate analysis (PCoA) of bacterial communities in fresh materials and triticale silage after 7 and 30 days of ensiling; FM, fresh matter CON, control (treated with distilled water); ST, inoculated with *S. bovis*; LP, inoculated with *L. plantarum*; LS, inoculated with *S. bovis* and *L. plantarum.*

**Fig 3:**
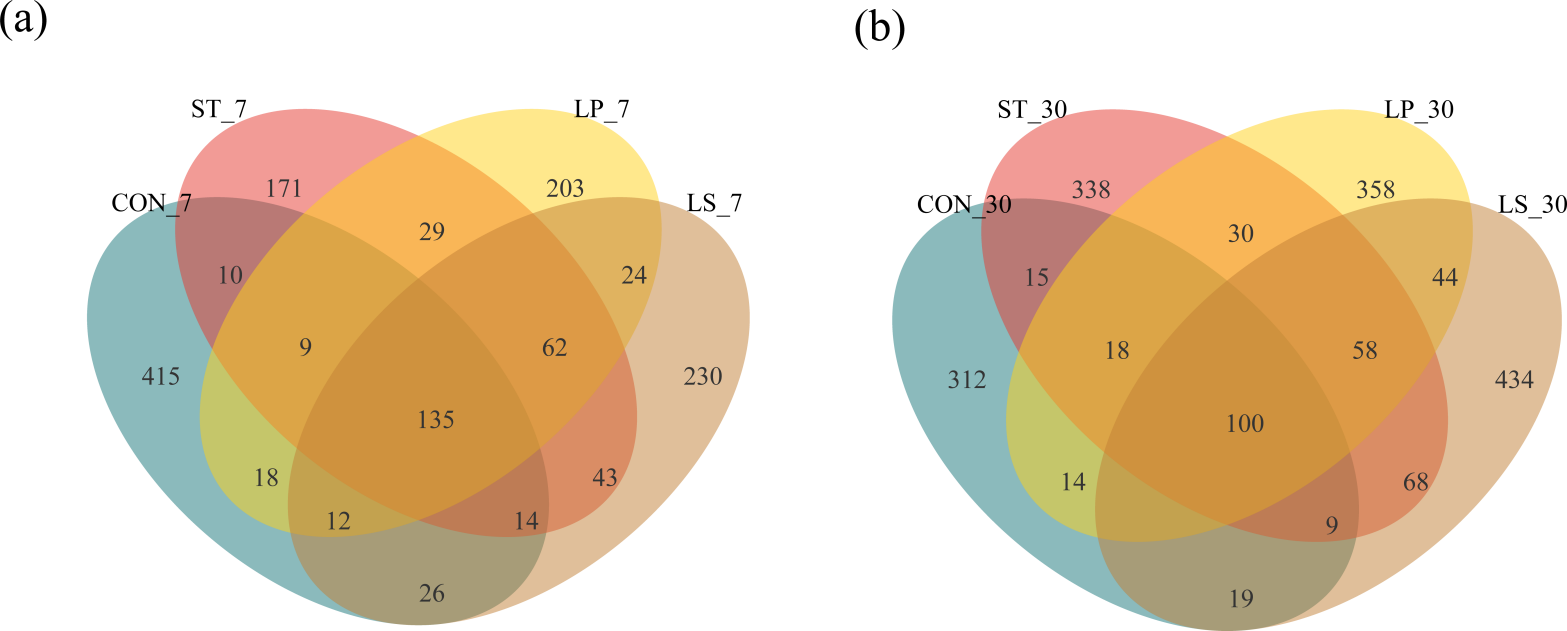
Venn diagrams of unique or shared bacterial ASVs in silage after 7 (**a**) and 30 days (**b**) of different treatments; CON, control (treated with distilled water); ST, inoculated with *S. bovis*; LP, inoculated with *L. plantarum*; LS, inoculated with *S. bovis* and *L. plantarum.*

Variance in the bacterial community at fresh, 7, and 30 days was observed using principal coordinates analysis (PCoA) at the ASV level (Fig. 2). PCo 1 and PCo 2 accounted for 35% and 23.2% of the total variance, respectively. The PCoA plot showed that the bacterial communities in the fresh, day 7, and day 30 silage samples were significantly different, indicating that fermentation day significantly altered the bacterial community structure. During ensiling, common ASVs decreased, whereas specific ASVs increased (except in the CON group). On day 7, the Venn analysis (Fig. 3a) revealed 135 common ASVs across all groups and 415, 171, 203, and 230 specific ASVs in the CON, ST, LP, and LS groups, respectively. On day 30 (Fig. 3b), all the groups contained 100 common ASVs, whereas the CON, ST, LP, and LS groups contained 312, 338, 358, and 434 ASVs, respectively.

**Fig 4:**
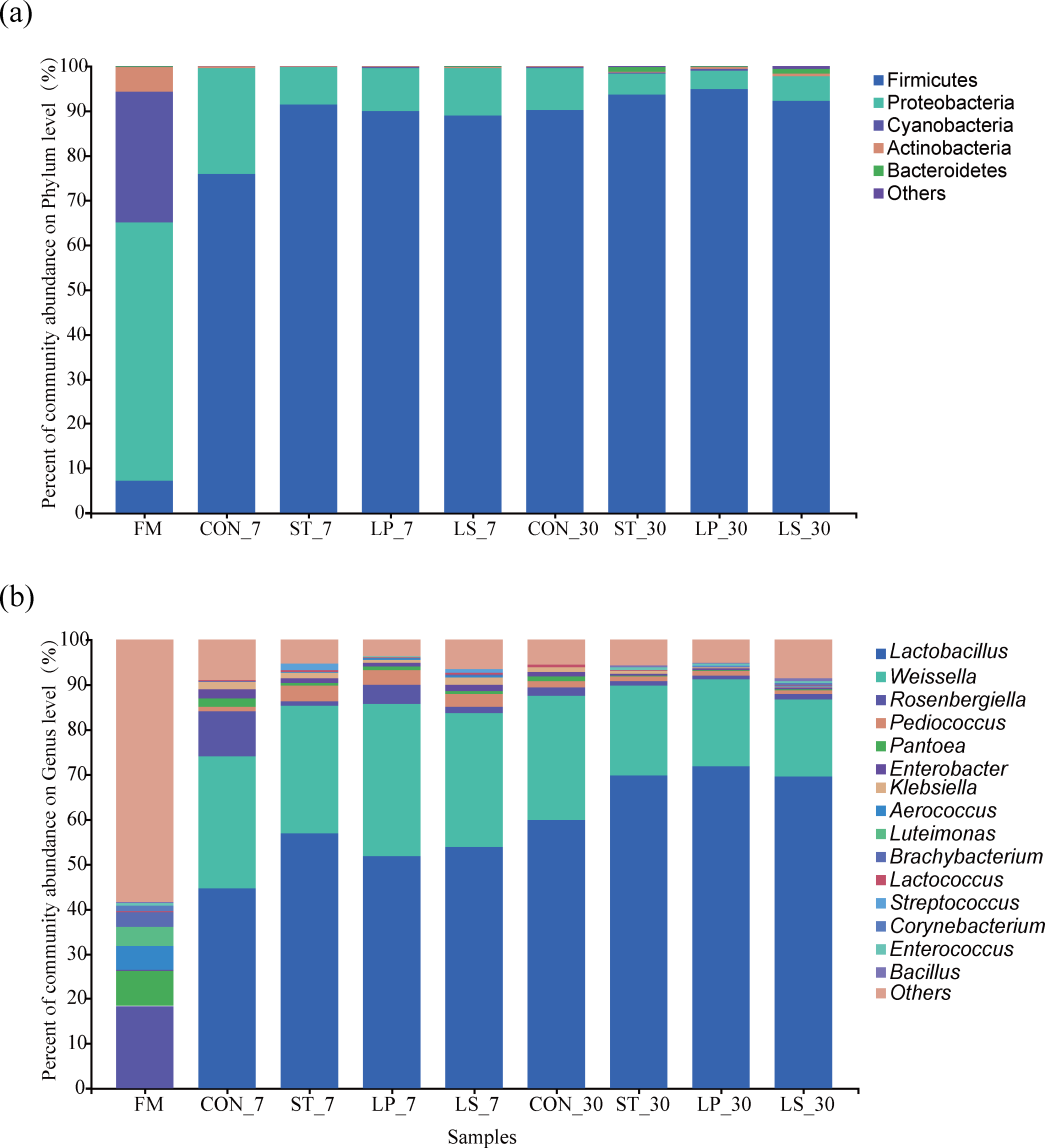
Bacterial community and relative abundance of different days of ensiled triticale samples at the phylum level (**a**) and genus level (**b**). FM, fresh matter CON, control (treated with distilled water); ST, inoculated with *S. bovis*; LP, inoculated with *L. plantarum*; LS, inoculated with *S. bovis* and *L. plantarum.*

The top 5 bacteria at the phylum level and the top 15 bacteria at the genus level in terms of silage abundance are shown in Fig. 4; Tables S4 and S5. Before ensiling, Proteobacteria was the predominant phylum (57.75%), followed by Cyanobacteria (29.24%), Firmicutes (7.31%), Actinobacteria (5.46%), and Bacteroidetes (0.19%). After ensiling, the abundance of Firmicutes in the inoculation group significantly increased (88.92%–91.43% vs 75.80%, *P* < 0.001), while the Proteobacteria (8.28%–10.60% vs 23.71%, *P* < 0.001) significantly decreased compared to the CON group. At the genus level, *Lactobacillus* (44.72%–71.89%) and *Weissella* (17.20%–38.41%) were the predominant genus in the silage, followed by *Rosenbergiella* (0.81%–10.11%), and *Pediococcus* (0.94%–3.60%). On day 7, the inoculated groups showed significantly increased abundances of *Lactobacillus* (*P* < 0.05) and *Pediococcus* (*P* = 0.006) but significantly reduced abundances of *Rosenbergiella* and *Enterobacter* (*P* < 0.001) compared to the CON group. Notably, the abundance of *Lactococcus* in the ST and LS groups was significantly higher than in the CON and LP groups (*P* = 0.001). Additionally, the abundance of *S. bovis* in the ST and LS groups was 1.32% and 0.78%, respectively; however, it was not detected in the CON and LP groups. On day 30, the abundance of *Lactobacillus* in the CON, ST, LP, and LS groups increased by 14.34%, 12.73%, 20.20%, and 15.77%, respectively, compared with that on day 7. The abundance of *Rosenbergiella*, *Pediococcus*, *Pantoea*, *Enterobacter*, *Klebsiella,* and S*treptococcus* decreased in all groups.

### Co-occurrence networks in the bacterial community

**Fig 5:**
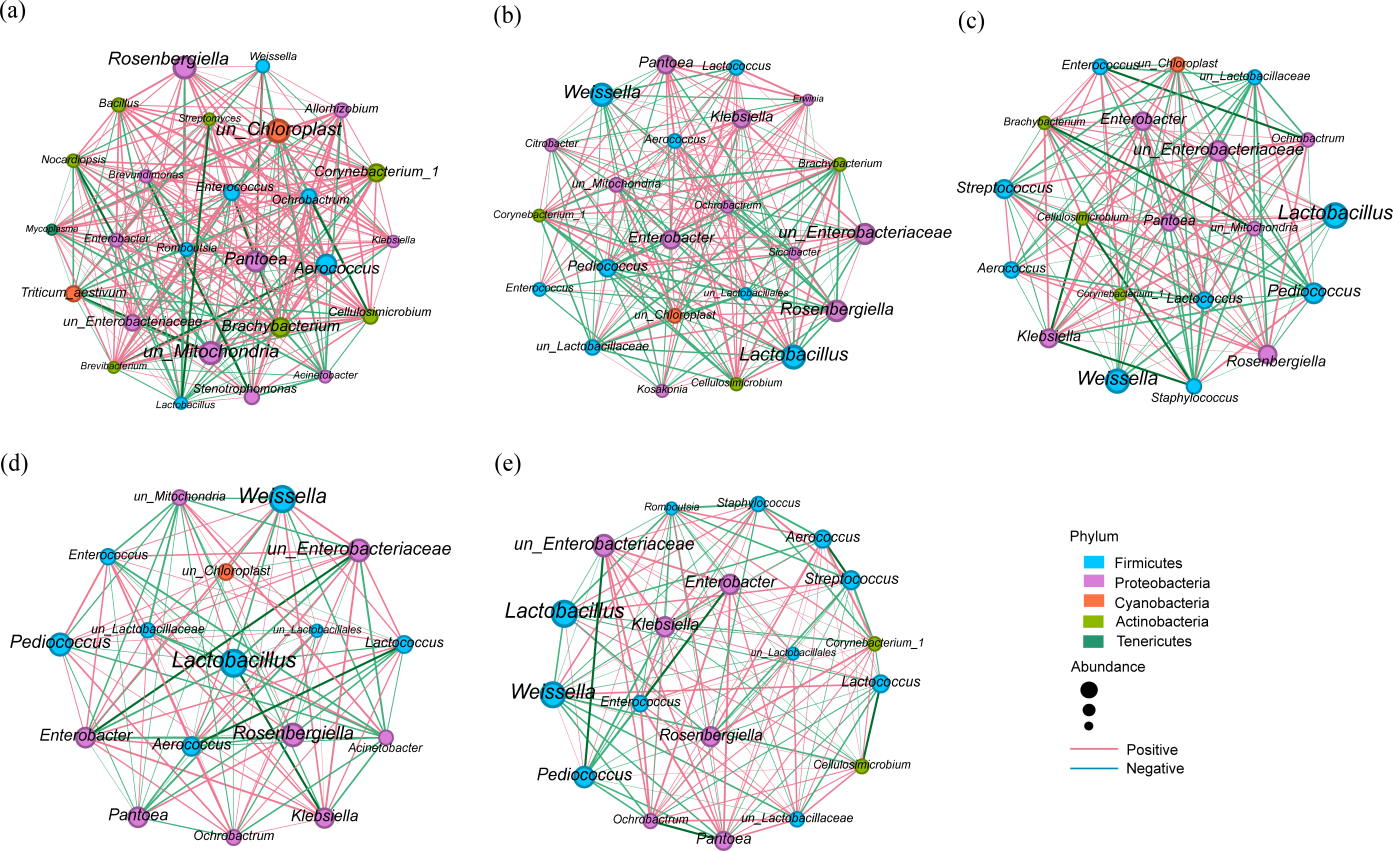
The bacterial symbiotic network of fresh and ensiled triticale (Spearman correlation, the most abundant 100 species, *P* < 0.05, and correlation > 0.5) of FM (a), CON (**b**), ST (**c**), LP (**d**), and LS (**e**).

**Fig 6:**
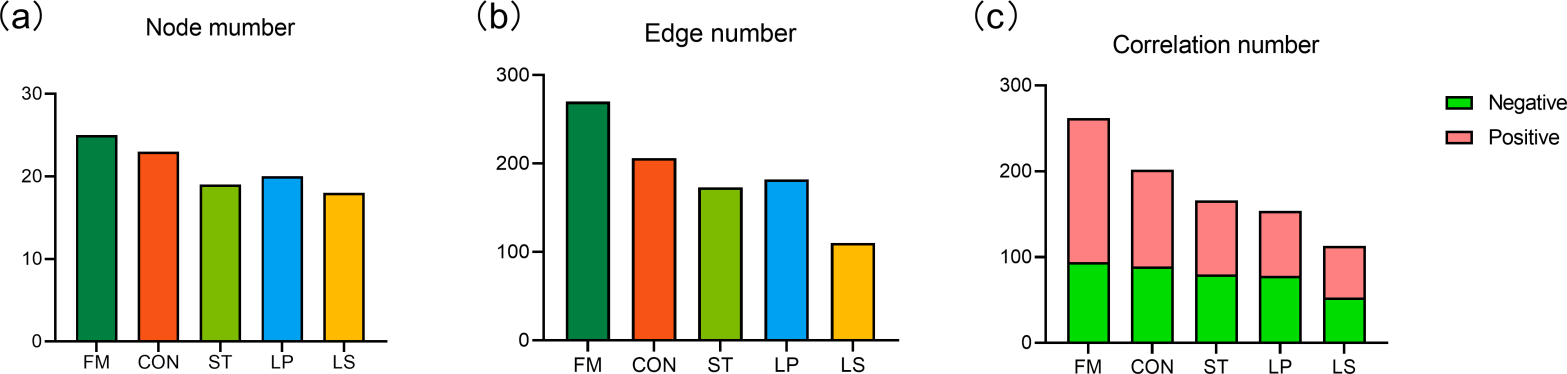
The bar chart node mumber (**a**), edge number (b), and correlation number (c). FM, fresh matter; CON, control (treated with distilled water); ST, inoculated with *S. bovis*; LP, inoculated with *L. plantarum*; LS, inoculated with *S. bovis* and *L. plantarum.*

The bacterial symbiotic networks of fresh and silage samples are shown in Fig. 5. The anaerobic fermentation process significantly simplified the structural complexity of the bacterial community, while the inoculation of LAB further reduced this complexity (Fig. 5a through e). The bar chart (Fig. 6) compares the node number, edge number, and correlation number of the bacterial co-occurrence network. In the silage, both edge number and node number were lower than in FM. Compared to single inoculation or non-inoculation treatments, the combined inoculation of *S. bovis* and *L. plantarum* resulted in a lower bacterial network complexity, which is consistent with the results of bacterial community diversity.

### Metabolic prediction of bacterial community analysis by PICRUSt 2

**Fig 7:**
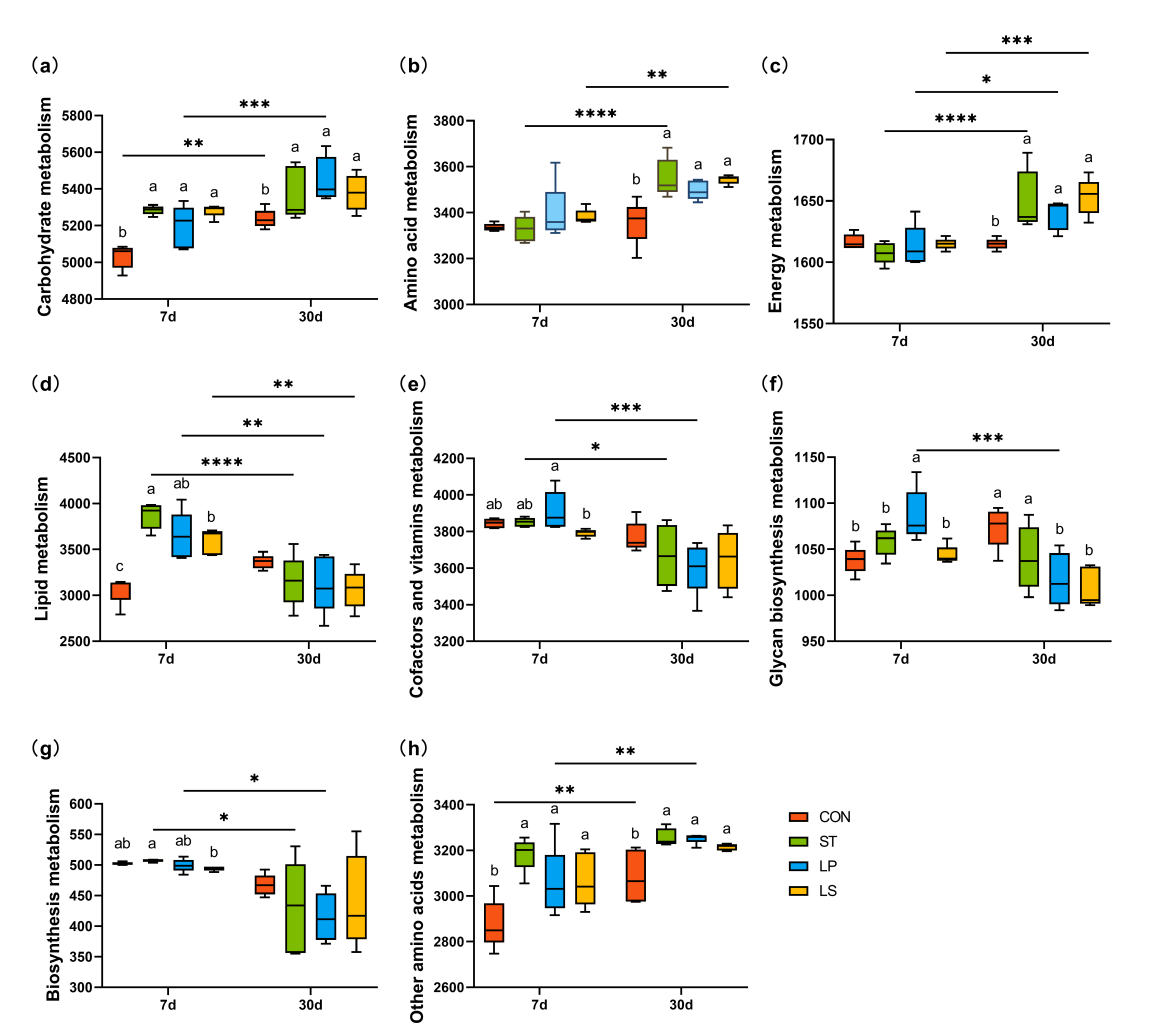
Functional prediction of bacterial level 2 metabolic pathways in triticale silage after 7 and 30 days. Values with different lowercase letters indicate significant differences among treatments on the same ensiling day (*P* < 0.05); asterisks indicate significant differences among ensiling days in the same treatment (*P < 0.05, **P < 0.01, ***P < 0.001); CON, control (treated with distilled water); ST, inoculated with *S. bovis*; LP, inoculated with *L. plantarum*; LS, inoculated with *S. bovis* and *L. plantarum.*

We utilized PICRUSt 2 software to predict bacterial metabolic functions during ensiling via KEGG pathways. As shown in Fig. S2, the relative abundance related to “metabolism” was greater than other pathways, with the relative abundance of carbohydrate metabolism being significantly higher than other pathways. Therefore, we focused on metabolism analysis. Box plots display the differences in bacterial community functions on days 7 and 30 after ensiling (Fig. 7). After 7 days of ensiling, the inoculation of LAB, the relative abundances of carbohydrate metabolism, lipid metabolism, and other amino acid metabolism significantly increased (*P* < 0.05), while the relative abundances of amino acid metabolism and energy metabolism showed a significant increased after 30 days of ensiling (*P* < 0.05). During the ensiling, the cofactors and vitamins metabolism, glycan biosynthesis and metabolism, and biosynthesis metabolism exhibited slight fluctuations.

**Fig 8:**
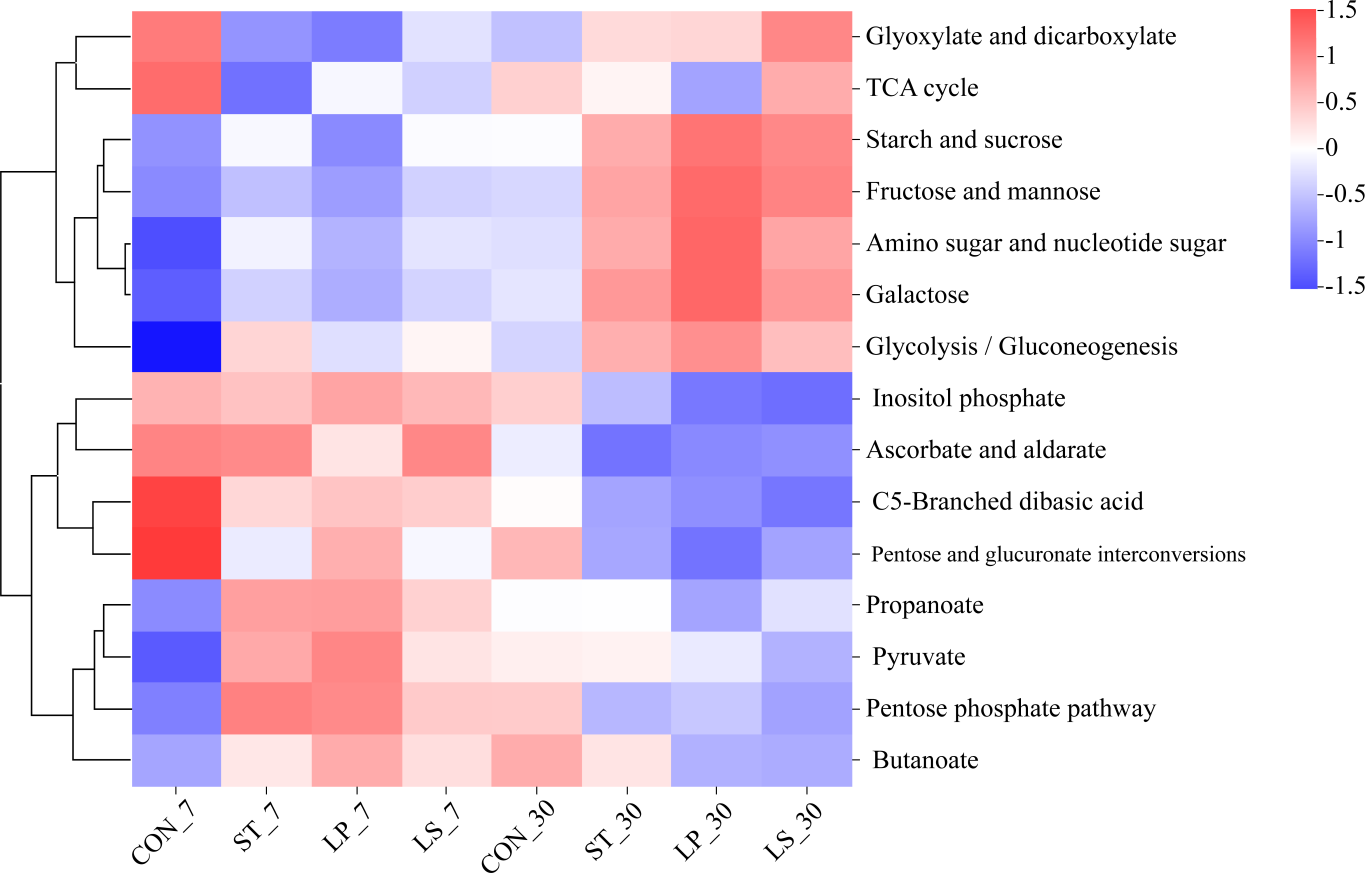
Functional prediction of Level 3 bacterial metabolic pathways involved in carbohydrate metabolism with triticale silage for 7 and 30 days; CON, control (treated with distilled water); ST, inoculated with *S. bovis*; LP, inoculated with *L. plantarum*; LS, inoculated with *S. bovis* and *L. plantarum.*

In addition, we analyzed carbohydrate metabolism on the level 3 pathway. As shown in Fig. 8, there was a significant difference between the groups with and without the inoculant. The abundance of starch and sucrose metabolism, amino sugar and nucleotide sugar metabolism, galactose metabolism, fructose and mannose metabolism, and glycolysis/gluconeogenesis metabolism across all groups increased with ensiling days. Conversely, the abundance of metabolites involved in ascorbate and aldarate metabolism, inositol phosphate metabolism, c5-branched dibasic acids metabolism, and pentose and glucuronate interconversion metabolism decreased with ensiling days. Inoculation with LAB enhanced the metabolism of propanoate, pyruvate, pentose phosphate pathway, and butanoate after 7 days of ensiling. After 30 days of ensiling, compared to the CON, the inoculation significantly increased the metabolism of starch and sucrose, amino sugar and nucleotide sugar, fructose and mannose, galactose, as well as glycolysis/gluconeogenesis. Notably, on day 7, the abundance of starch and sucrose, amino sugar and nucleotide sugar, and galactose metabolism was higher in the ST and LS groups than in the CON and LP groups.

### Correlation analysis between carbohydrate content, fermentation characteristics, and bacterial community

**Fig 9:**
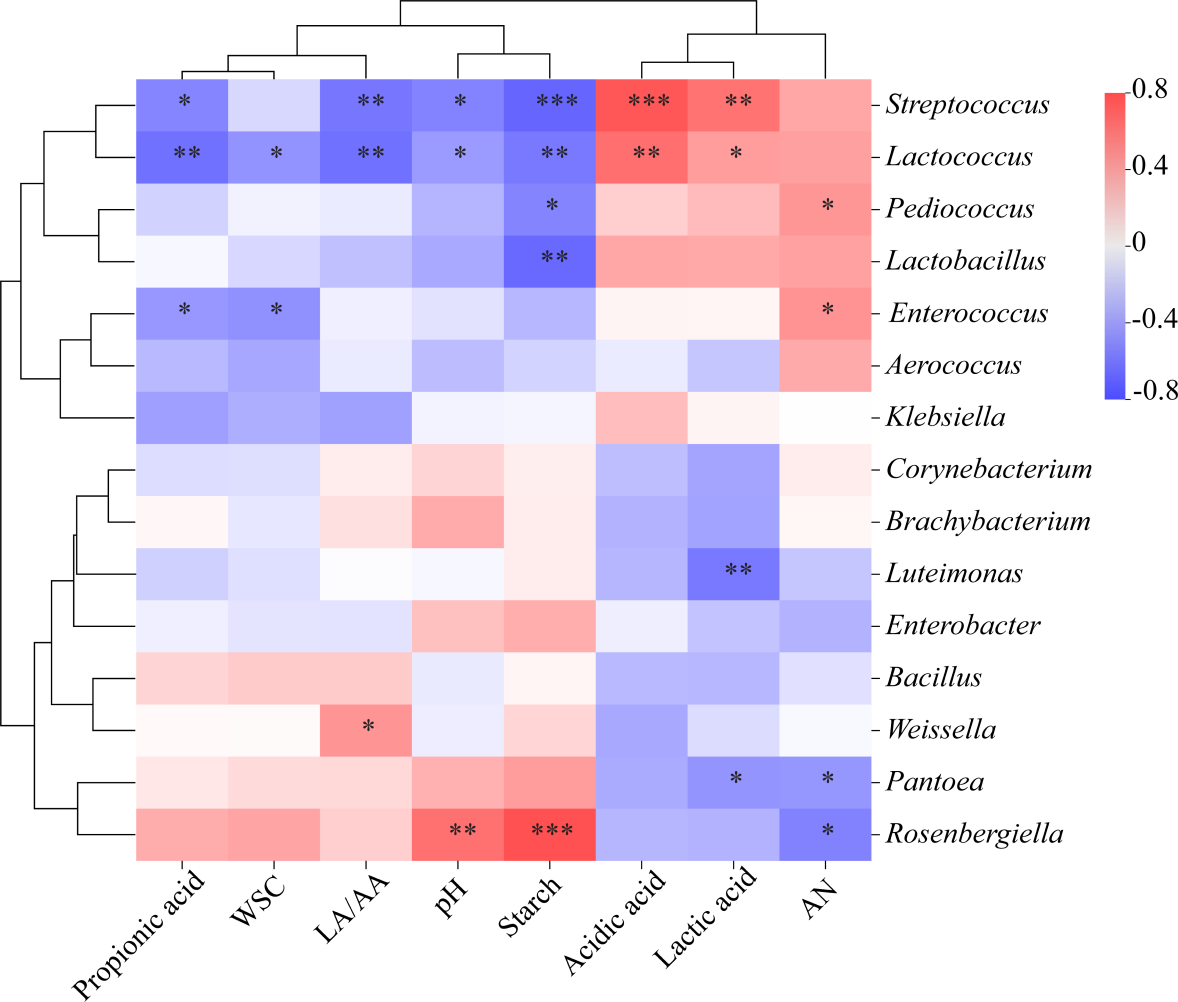
Correlation analysis of carbohydrate content, fermentation characteristics, and bacterial community with triticale silage for 7 days. Asterisks indicate significant differences (*P < 0.05, **P < 0.01, ***P < 0.001).

Our results indicate that *S. bovis* may play a more positive role in the early stages of silage production than in later stages. Thus, to assess the carbohydrate hydrolysis in triticale silage, we conducted a cluster correlation analysis between starch, WSC, fermentation characteristics, and the top 15 bacterial genera on day 7. Fig. 9 shows that the abundance of *Lactococcus* and *Streptococcu*s are significantly positively correlated with LA and AA, while they are significantly negatively correlated with starch, pH, the Lactic acid to acetic acid ratio (LA/AA), and PA. *Lactobacillus* and *Pediococcus* showed significant negative correlations with starch. Furthermore, *Rosenbergiella* exhibited a significant positive correlation with starch and pH but a significant negative correlation with AN concentration. *Luteimonas* and *Pantoea* showed significant negative correlations with LA concentrations.

## DISCUSSION

### Variation in the chemical composition of triticale silage

Triticale, rich in starch, protein, and fiber, offers high yields and shows rapid growth and drought resistance. However, its hollow stem makes it susceptible to aerobic deterioration (26). Appropriate additives can improve the triticale silage quality and enhance livestock production.

Cereal crop silage is widely used as a livestock feed because it is rich in starch and cellulose. To our knowledge, LAB does not utilize starch during silage fermentation. However, this study found that the starch content in the groups treated with *S. bovis* was reduced compared to the groups treated with *L. plantarum* (which showed a decrease of 4.98%–6.58%) and the control group (CON, which showed a decrease of 9.16%–10.82%) in the early stages of ensiling. This difference might be because the α-amylase secreted by *S. bovis* acts on starch molecules, cutting the glycosidic bonds through hydrolysis, breaking starch into smaller sugar molecules, and converting insoluble starch into soluble sugar (27, 28). In contrast, Jones et al. demonstrated that starch in alfalfa silage was not hydrolyzed by *S. bovis* as expected (10). This may be due to the low starch content in alfalfa (1%–3%), which might be insufficient to support the growth and metabolic activity of *S. bovis*. In contrast, the higher starch content in triticale (12%–15%) likely provides a richer substrate for *S. bovis*, resulting in significant consequences in the statistical analysis.

In addition, *S. bovis* and *L. plantarum* had a synergistic effect that enabled them to utilize WSC and rapidly reduce the pH value. In line with this, several studies have demonstrated that compared with LAB-based silage, the use of *S. bovis* results in a reduction of 30% or more in the doubling time (28, 29), leading to faster pH reduction in silage, similar to *Enterococcus faecium* (10). Previous studies have shown that LAB additives reduce DM and CP loss (30, 31). In the present study, LAB treatment resulted in less DM loss than in the CON group. This difference may be attributed to swift fermentation leading to rapid pH reduction, inhibiting the growth of detrimental bacteria such as *Enterobacterium* and *Clostridium*. Furthermore, LAB produce antimicrobial substances such as organic acids, hydrogen peroxide, and bacteriocins, which inhibit the growth of harmful bacteria (32).

### Variation in the fermentation characteristics of triticale silage

LAB produces a significant amount of LA during ensiling, thus reducing the pH (33). Consequently, pH is a critical parameter for evaluating silage fermentation efficiency (34). In this study, the pH values of all groups decreased below 4.0, suggesting that fresh triticale could be effectively fermented and preserved with or without the inoculant. This may be due to the high WSC content, low buffering capacity, and epiphytic LAB of the raw materials (35). Nevertheless, the ST and LS groups inoculated with *S. bovis* exhibited higher LA production and lower pH during the early stages of ensiling. Generally, for silage, lower pH values result in higher LA production (34, 35). This result is verified in the present study. The higher LA concentration in the inoculated group could be due to the shorter time and greater number of LAB required to produce organic acids during the early stages. All groups underwent homofermentative fermentation (LA/AA > 3.0) (36). On days 3 and 7, the LA and AA concentrations were higher in the ST and LS groups, but their LA/AA ratios were significantly lower than those in the CON and LP groups. In contrast, Zhao et al. (31) reported that *Leuconostoc lactis* and *Weissella confusa* had relatively high LA/AA ratios during ensiling. The reason for this discrepancy could be that *S. bovis* functions as a quick starter during fermentation, encouraging the growth of homofermentative LAB (producing LA) at the beginning of fermentation, while accelerating the growth of heterofermentative LAB (producing AA). However, with decreasing pH, the growth of *S. bovis* was inhibited. This also explains why AA production and LA/AA ratio converged at a later stage. Typically, PA and BA are undesirable products in silage because their production results in energy wastage, and the PA content of high-quality silage should be between 1 and 10 g/kg DM (37, 38). In the present study, PA concentrations were low across all groups (1.06–7.50 g/kg DM), and BA was undetected. This also indicated no secondary fermentation during ensiling, indicating the excellent quality of silage fermentation. Notably, the PA concentrations in the ST and LS groups with *S. bovis* were lower than those in the CON and LP groups. This may be attributed to the involvement of *S. bovis*, which inhibits the growth of some PA-producing bacteria, and its low pH, which restricts the conversion of lactate to propionate. *Clostridia* decompose proteins to produce AN, which results in poor fermentation quality. However, our study did not detect BA, indicating that harmful bacteria such as *clostridia* were inhibited. Some LABs produce proteases during ensiling, which further generate AN via protein degradation (39, 40). In this study, *S. bovis* may have facilitated the growth of other LABs, which enhanced the production of proteases by these bacteria. This resulted in the treatment with *S. bovis* which had higher levels of AN during the early stages of ensiling. This may explain why the LS group had the highest LA concentration and lowest pH but still had a higher AN concentration in the early stage.

### Variation in the bacterial community of triticale silage

Previous studies have elucidated the role of *S. bovis* in silage by analyzing its chemical properties and fermentation characteristics (10, 11, 41). The 16S rRNA sequencing technology can reveal bacterial diversity and dynamic variations more accurately and comprehensively than traditional methods. To our knowledge, this is the first study to use NGS technology to assess the effects of *S. bovis* in silage on bacterial communities and to predict variations in metabolic function. The coverage of fresh triticale and silage was more than 0.99, indicating that most of the target gene regions for sequencing were read and covered, thereby ensuring data integrity and accuracy (42). Bacterial diversity is generally lower in low pH value silage, and pH largely determines the bacterial α-diversity (43). Similar results were observed in our study, where inoculation with LAB reduced α-diversity compared to CON, and the combined inoculation of *L. plantarum* and *S. bovis* further intensified this effect. This may be attributed to the rapid proliferation of *S. bovis* and the antibacterial properties of *L. plantarum*, which inhibit undesirable bacterial growth (10, 44).

Additionally, combining the results of the PCoA and Venn plots further explained the impact of bacterial community differences between the treatments on ecological functionality and environmental adaptability. The PCoA results revealed that anaerobic fermentation significantly influenced the bacterial composition of silage, leading to a distinct separation among all groups during the early ensiling stage. When compared to CON, the Venn plots indicated that the unique ASVs were reduced in all treatments on day 7 but increased on day 30; conversely, common ASVs decreased during ensiling. This could be attributed to the promotion of LA production by inoculation in the early stages, creating an acidic environment that inhibits the growth of aerobic and other undesirable bacteria, further facilitating the proliferation of LAB in the later stages (26).

Bacterial communities play a crucial role in the silage fermentation quality (26). In this study, we observed a clear succession of Proteobacteria to Firmicutes before and after fermentation, which is consistent with the literature (26, 30, 31). This can be mainly attributed to the acidic environment, which suppresses the growth of acid-sensitive bacteria (*Enterococcus*, *Staphylococcus*, yeast, mold, etc.) while promoting the proliferation of LAB. Moreover, Firmicutes can degrade complex organic compounds found in silage, including starch, proteins, and cellulose (45). In the present study, the ST group (day 7) showed the highest abundance of Firmicutes. In addition, *S. bovis* increased starch hydrolysis, resulting in more WSC for Firmicutes utilization and further increasing its abundance.

At the genus level, *Rosenbergiella*, *Enterobacter*, and *Pantoea* were the predominant genera in fresh triticale, which is consistent with the results of previous studies (46, 47). *Rosenbergiella* and *Pantoea* compete with LAB for sugars, while *Enterobacter* generates ammonia and other putrefaction products that hinder fermentation, like hydrogen sulfide and biogenic amines. After ensiling, the abundance of *Rosenbergiella*, *Enterobacter,* and *Pantoea* significantly decreased and was replaced by LAB. This can be attributed to the anaerobic and acidic environment, which limited the growth of these undesirable bacteria. LAB remain unaffected because of their vital acid tolerance, and organic acids produced by LAB proliferation further reduce the pH and inhibit undesirable bacteria (48). Additionally, *S. bovis* produces antibacterial substances, such as organic acids and bovicin HC5 (10, 13), which suppress undesirable bacteria in silage.

*Weissella*, *Lactococcus*, and *Pediococcus* are commonly encountered during the initial stages of silage fermentation (49). Yang et al. (50) reported that with pH reduction, acid-tolerant *Lactobacilli* replaced *Weissella, Pediococcus, and Lactococcus* during ensiling, which is similar to the results of our study. In this study, inoculation with *S. bovis* and *L. plantarum* increased the proportion of *Pediococcus* and *Lactococcus* in silage on day 7 compared to that in the CON group. On day 30, the CON group had significantly higher *Weissella* and *Lactococcus* abundance than the other groups. This may be because the inoculant promotes LAB growth during the early stages, whereas the CON group without additives exhibits slower fermentation. Additionally, the abundance of *S. bovis* decreased significantly over time, which was consistent with the changes in *Lactococcus* and *Pediococcus*. This suggests that *S. bovis* rapidly initiates fermentation during the early stages to create favorable conditions for other LAB; however, its growth is gradually inhibited as the pH decreases.

### Variation in bacterial metabolism of triticale silage

Microorganisms convert substrates into various metabolites via complex metabolic pathways that affect silage fermentation quality (37). KEGG analysis showed that most of the metabolic pathways were involved in metabolism, indicating that metabolism may be the most critical factor influencing triticale silage fermentation. At level 2 metabolic pathways, carbohydrate, amino acid, energy, and other amino acid metabolic pathways were upregulated after 30 days of ensiling. A previous study showed a significant positive correlation between the metabolism of amino acids and carbohydrates and the main products of high-quality silage (51). Amino acid decarboxylation, malate decarboxylation, and arginine deamination are the three main energy metabolic pathways involved in LA accumulation during LAB fermentation (52). These upregulated metabolic pathways indicated that bacteria (presumably *Lactobacillus*) remained active, breaking down polysaccharides into monosaccharides and LA. Correspondingly, the upregulated groups exhibited a higher level of *Lactobacillus* abundance and LA production in this study. However, this result contradicts the findings of Wang et al. (53), who reported that energy metabolism decreases during ensiling. Therefore, further study is needed to investigate the impact of anaerobic fermentation on energy metabolism. Nutrient loss can occur owing to respiration, proteolysis, and lipolysis during the early stages of ensiling. Proper ensilage techniques help maintain the nutrient content by minimizing losses due to spoilage and respiration (54). In the present study, lipid metabolism pathways were downregulated on day 30 compared to day 7. This may be because plant matter is primarily fermented by LAB, which converts sugars into LA, thus lowering pH, inhibiting undesirable bacteria, and preserving nutrients, including lipids, in the silage (55). Furthermore, our results indicated that the CON group showed significantly lower levels of upregulated pathways and significantly higher levels of downregulated pathways than the inoculant-treated group. This was consistent with the higher pH, lower lactate production, and lower LAB abundance observed in the CON group. The lack of additional LAB may explain the delayed fermentation, lower LA production, and higher pH values in the CON group.

To investigate the utilization of carbohydrates in silage using additives, we analyzed carbohydrate metabolism. During ensiling, starch and sucrose provide energy, and their metabolism produces organic acids such as LA, which are crucial for lowering silage pH and preventing the accumulation of spoilage organisms (56). Amino sugar and nucleotide sugar metabolism produce intermediates for synthesizing cellular components, including those that improve silage quality, such as 3-phosphoglycerate, amino acids, and coenzyme A (54). Interestingly, on day 7, the group inoculated with *S. bovis* showed significant upregulation of these two metabolic pathways in the inoculated group. Similar results were obtained for glycolysis/gluconeogenesis and galactose metabolism. Upregulation of these metabolic pathways indicates that microorganisms, particularly *S. bovis*, actively ferment sugars to produce LA and other organic acids. This is consistent with the fermentation and bacterial composition results. However, the functional changes of the bacterial community predicted were based on phylogenetic inference from 16S rRNA in this study, and other omics approaches (such as proteomics and metabolomics) should be applied to further assess the bacterial community’s functions during the ensiling.

### Correlation between carbohydrate content, fermentation characteristics, and microflora

Correlations between bacterial composition and various chemical indicators provide a deeper understanding of the fermentation mechanisms of silage and the microbiological basis of its quality. During ensiling, the interaction between the chemical composition and microorganisms affects the metabolic products of the final silage. These parameters are positively correlated with beneficial bacteria and negatively correlated with harmful bacteria (57). In this study, *Streptococcus* and *Lactococcus* positively affected pH, LA, AA, and LA/AA but negatively affected PA. Our results indicate that the abundance of *Lactococcus* and *Streptococcus* in the ST and LS groups was significantly higher than in the CON and LP groups in the early fermentation phase. Additionally, LA concentrations in the ST and LS groups were elevated on days 3 and 7, while pH values decreased on day 7. However, after 30 days of ensiling, there were no significant differences in the abundance of *Lactococcus* and *Streptococcus* between the inoculated groups, and their abundance significantly declined. Therefore, we suggest that *Lactococcus* and *Streptococcus* may play a more critical role in pH changes during the early stage of ensiling, consistent with previous studies (58, 59). PA is undesirable for fermentation because of its potential energy wastage (37).

Typically, *Lactobacillus* dominates silage fermentation and positively affects pH and organic acids (6, 54). However, this study found no significant correlations between *Lactobacillus* and WSC, pH, LA, or AA levels. This may be because of the ecological niche overlap between *Lactobacillus* and *Streptococcus*, resulting in no statistically significant correlation with *Lactobacillus.* Despite potential niche overlap, the two genera did not exhibit competitive exclusion in our study. Instead, their co-presence resulted in mutual benefits that improved fermentation quality. This suggests that niche overlap between *Lactobacillus* and *Streptococcus* in silage fermentation can lead to synergistic rather than competitive interactions. The synergistic effects between *Lactobacillus* and *Streptococcus* are demonstrated by the enhanced fermentation quality of the silage when both bacteria are present. These effects are reflected in improved fermentation characteristics, beneficial shifts in bacterial community dynamics, and significant correlations with key fermentation indicators.

In addition, we found a significant negative correlation between *Pantoea* and AN, consistent with the findings of Ogenade et al., who reported that an increased *Pantoea* abundance could reduce AN concentrations in silage (49). However, the specific mechanism through which *Pantoea* degrades AN remains unclear and requires further investigation.

### Synergistic effects of lactic acid bacterial inoculants

At the present study, we observed that the synergistic interaction between *S. bovis* and *L. plantarum* significantly enhanced the fermentation quality of triticale silage. This synergy can be explained by several factors. First, *S. bovis* proliferates rapidly in the early fermentation stage, promoting lactic acid production and lowering pH, thereby creating an acidic environment conducive to the growth of other LABs, such as *L. plantarum*. This finding aligns with previous studies on co-inoculants, indicating that interactions among different microorganisms can significantly influence fermentation products and community dynamics (49, 60). Additionally, the antimicrobial properties of *L. plantarum* inhibit the growth of undesirable bacteria, further enhancing the fermentation efficacy of *S. bovis*. We found that the co-inoculation group exhibited higher lactic acid concentrations and lower pH values in the early stages of ensiling, indicating that their interaction promotes beneficial bacteria proliferation while suppressing harmful bacteria.

Furthermore, co-inoculation led to significant changes in bacteria community structure, increasing the abundance of LAB and reducing that of undesirable bacteria (e.g., *Pantoea* and *Enterobacter*). These dynamic shifts in the bacteria community further support our hypothesis regarding the synergistic effect between *S. bovis* and *L. plantarum*. Therefore, we recommend further investigation into the synergistic effects of different microbial combinations in future research to optimize the fermentation process of ensiling and enhance its nutritional value.

### Conclusion

Inoculation with *S. bovis* effectively improved the fermentation quality, such as LA and AA content, in triticale silage by increasing the relative abundance of LAB and reducing the relative abundance of *Pantoea*, *Enterobacter*, and *Weissella. S. bovis* initiated rapid silage fermentation at the early stages. Co-inoculated with *S. bovis* and *L. plantarum* further accelerated pH reduction and increased LA production. Therefore, *S. bovis* can be used as an additive or for fast start-up agent to improve silage fermentation quality.

## Data Availability

The raw sequencing data have been deposited in NCBI’s Sequence Read Archive (SRA) under the accession number PRJNA1122775.

## SUPPLEMENTAL MATERIAL

Fig. S1 msphere.00894-24-s0001.docx Hemolysis assay of *S. bovis*.

Fig. S2 msphere.00894-24-s0002.docx Abundance statistics of level 1 metabolic pathways triticale silage by KEGG.

Table S1 msphere.00894-24-s0003.docx Antimicrobial susceptibility testing of *S. bovis*.

Table S2 msphere.00894-24-s0004.docx Acute toxicity test results in mice.

Table S3 msphere.00894-24-s0005.docx Acute toxicity test data for mice.

Table S4 msphere.00894-24-s0006.xls Microbial composition of triticale silage at the phylum level.

Table S5 msphere.00894-24-s0007.xls Microbial composition of triticale silage at the genus level.

ASM does not own the copyrights to Supplemental Material that may be linked to, or accessed through, an article. The authors have granted ASM a non-exclusive, world-wide license to publish the Supplemental Material files. Please contact the corresponding author directly for reuse.
