# Supplementary material for: Fermentative profile and bacterial community structure of whole-plant triticale silage (Triticosecale Wittmack) with or without the addition of Streptococcus bovis and Lactiplantibacillus plantarum
Source: mSphere. 2025 Jan 28;10(2):e00894-24. doi: 10.1128/msphere.00894-24 (PMC11852913; doi:10.1128/msphere.00894-24)
Supplement: Fig. S1 — Hemolysis assay of S. bovis. [file msphere.00894-24-s0001.docx]

| 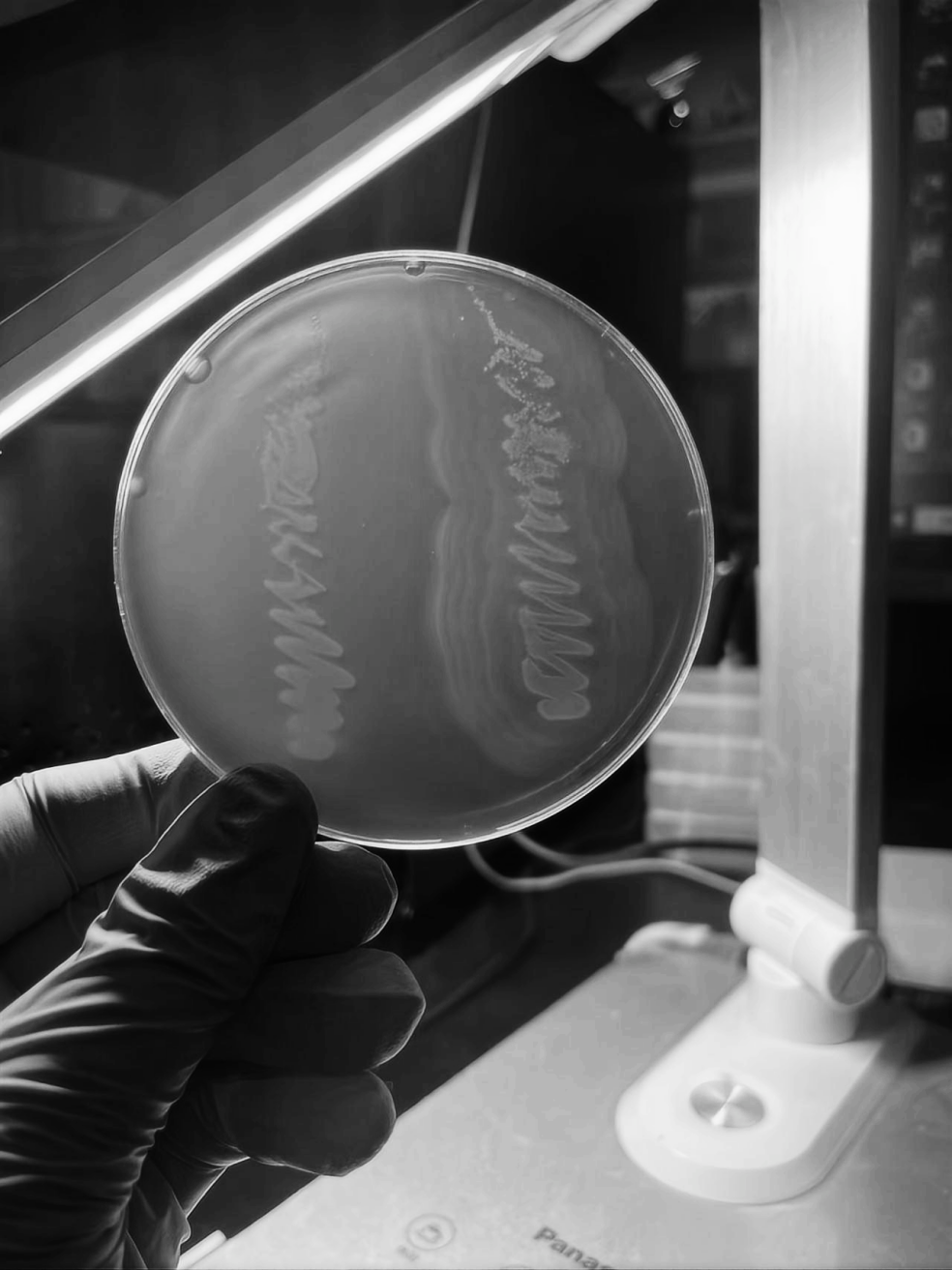 |
| --- |
|  |
| Fig.1 Hemolysis assay of *S. bovis*. The left and right display *S. bovis* and the positive control (*Staphylococcus aureus*), respectively. |
