# Supplementary figures and images for: Fermentative profile and bacterial community structure of whole-plant triticale silage (Triticosecale Wittmack) with or without the addition of Streptococcus bovis and Lactiplantibacillus plantarum
Source: mSphere. 2025 Jan 28;10(2):e00894-24. doi: 10.1128/msphere.00894-24 (PMC11852913; doi:10.1128/msphere.00894-24)

| 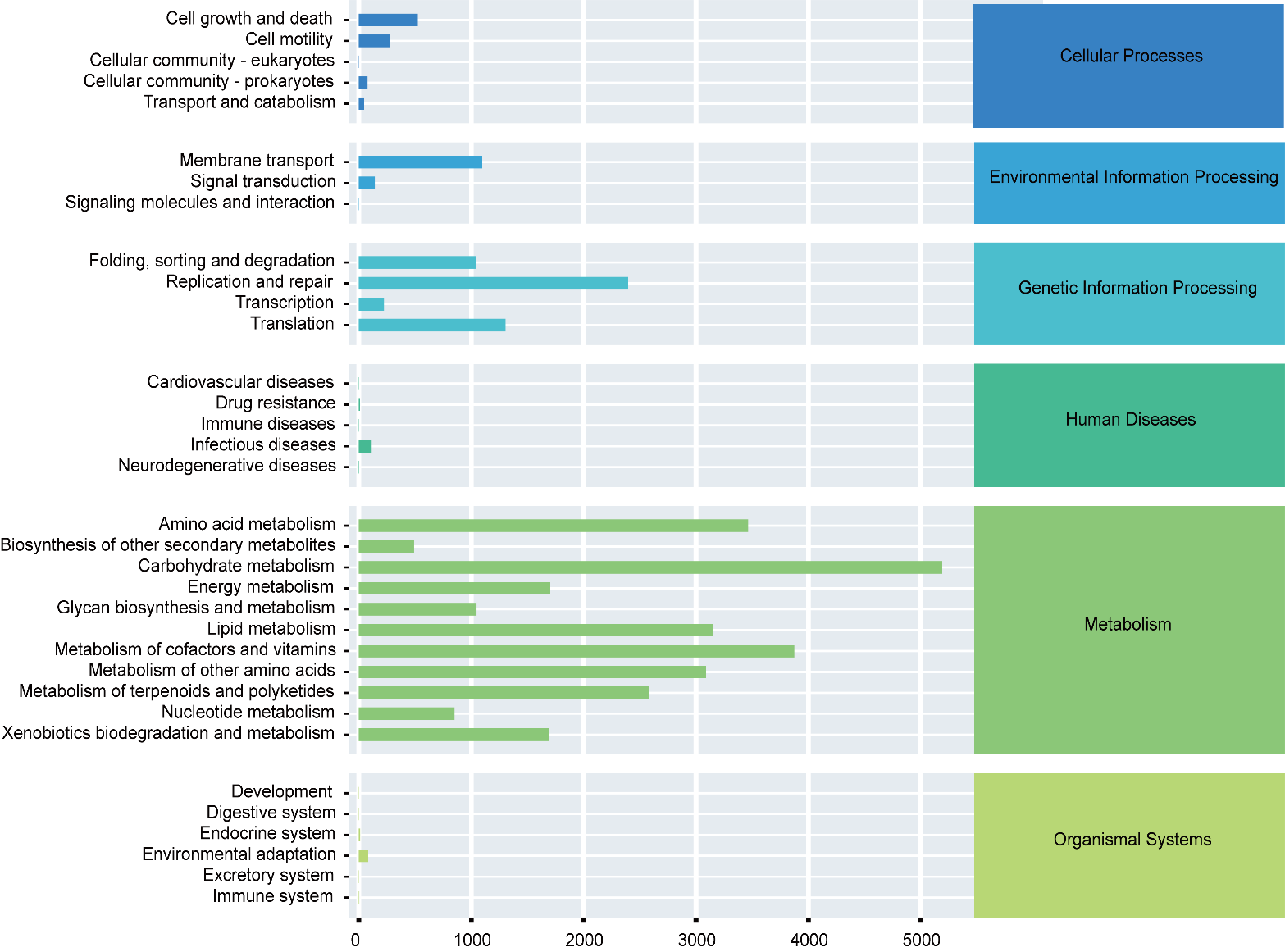 |
| --- |
| **Fig. S2** Abundance statistics of Level 1 metabolic pathways triticale silage by KEGG. |

Supplement: Fig. S2 — Abundance statistics of level 1 metabolic pathways triticale silage by KEGG. [file msphere.00894-24-s0002.docx]
