# Supplementary material for: Fermentative profile and bacterial community structure of whole-plant triticale silage (Triticosecale Wittmack) with or without the addition of Streptococcus bovis and Lactiplantibacillus plantarum
Source: mSphere. 2025 Jan 28;10(2):e00894-24. doi: 10.1128/msphere.00894-24 (PMC11852913; doi:10.1128/msphere.00894-24)
Supplement: Table S1 — Antimicrobial susceptibility testing of S. bovis. [file msphere.00894-24-s0003.docx]

| Table S1. Antimicrobial Susceptibility Testing of *S. bovis* | | |
| --- | --- | --- |
| Drug Name | Zone of Inhibition, mm | Resistance |
| Vancomycin | 21.53 | S |
| Clindamycin | 32.58 | S |
| Erythromycin | 35.44 | S |
| Rifampin | 34.54 | S |
| Gentamicin | 28.11 | S |
| Polymyxin B | 17.77 | S |
| Chloramphenicol | 28.29 | S |
| Amikacin | 24.57 | S |
| Enrofloxacin | 16.61 | I |
| Cefalotin | 16.29 | I |
| Neomycin | 19.46 | I |
| Levofloxacin | 9.29 | R |
| Tetracycline | 16.54 | R |
| Note: Sensitive (S), Intermediate (I), Resistant (R); | | |
