# Supplementary material for: Fermentative profile and bacterial community structure of whole-plant triticale silage (Triticosecale Wittmack) with or without the addition of Streptococcus bovis and Lactiplantibacillus plantarum
Source: mSphere. 2025 Jan 28;10(2):e00894-24. doi: 10.1128/msphere.00894-24 (PMC11852913; doi:10.1128/msphere.00894-24)
Supplement: Table S2 — Acute toxicity test results in mice. [file msphere.00894-24-s0004.docx]

| Table S2. Acute toxicity test results in mice | | |
| --- | --- | --- |
| Items | CON | EXP |
| Central Nervous System | No tremors, normal stress response | No tremors, normal stress response |
| Respiratory System | Normal respiration | Normal respiration |
| Gastrointestinal System | Stools are formed and of normal color | Stools are formed and of normal color |
| Reproductive and Urinary System | No contamination | No contamination |
| Skin and Fur | Shiny | Shiny |
| Eyes | No turbidity | No turbidity |
| CON, control group; EXP, experimental group. | | |
