# Supplementary material for: Fermentative profile and bacterial community structure of whole-plant triticale silage (Triticosecale Wittmack) with or without the addition of Streptococcus bovis and Lactiplantibacillus plantarum
Source: mSphere. 2025 Jan 28;10(2):e00894-24. doi: 10.1128/msphere.00894-24 (PMC11852913; doi:10.1128/msphere.00894-24)
Supplement: Table S3 — Acute toxicity test data for mice. [file msphere.00894-24-s0005.docx]

| Table S3. Acute toxicity test data for mice (n=5) | | | | |
| --- | --- | --- | --- | --- |
| Items | Male | | Female | |
|  | CON | EXP | CON | EXP |
| Initial weight, g | 26.58±3.13a | 26.49±2.68a | 24.76±2.33a | 25.14±1.88a |
| Final weight, g | 32.28±2.67a | 33.18±3.12a | 27.51±3.33b | 27.99±2.38b |
| Average intake, g/d | 8.22±1.76a | 8.02±0.53a | 6.52±0.36b | 6.76±0.53b |
| MTD, g/kg of BW | >20.0 | >20.0 | >20.0 | >20.0 |
| Mortality, % | 0 | 0 | 0 | 0 |
| CON, control group; EXP, experimental group; MTD, maximum tolerated dose. | | | | |
